# Assessing the Role of Calmodulin’s Linker Flexibility in Target Binding

**DOI:** 10.3390/ijms22094990

**Published:** 2021-05-08

**Authors:** Bin Sun, Peter M. Kekenes-Huskey

**Affiliations:** Department of Cell and Molecular Physiology, Loyola University Chicago, Maywood, IL 60153, USA; bsun@luc.edu

**Keywords:** calmodulin, coarse-grained molecular dynamics simulations, association kinetics

## Abstract

Calmodulin (CaM) is a highly-expressed Ca${}^{2+}$ binding protein known to bind hundreds of protein targets. Its binding selectivity to many of these targets is partially attributed to the protein’s flexible alpha helical linker that connects its N- and C-domains. It is not well established how its linker mediates CaM’s binding to regulatory targets yet. Insights into this would be invaluable to understanding its regulation of diverse cellular signaling pathways. Therefore, we utilized Martini coarse-grained (CG) molecular dynamics simulations to probe CaM/target assembly for a model system: CaM binding to the calcineurin (CaN) regulatory domain. The simulations were conducted assuming a ‘wild-type’ calmodulin with normal flexibility of its linker, as well as a labile, highly-flexible linker variant to emulate structural changes that could be induced, for instance, by post-translational modifications. For the wild-type model, 98% of the 600 simulations across three ionic strengths adopted a bound complex within 2 μs of simulation time; of these, 1.7% sampled the fully-bound state observed in the experimentally-determined crystallographic structure. By calculating the mean-first-passage-time for these simulations, we estimated the association rate to be $k_{a}=$ 8.7 × 10${}^{8}$ M${}^{-1}$ s${}^{-1}$, which is similar to the diffusion-limited, experimentally-determined rate of 2.2 × 10${}^{8}$ M${}^{-1}$ s${}^{-1}$. Furthermore, our simulations recapitulated its well-known inverse relationship between the association rate and the solution ionic strength. In contrast, although over 97% of the labile linker simulations formed tightly-bound complexes, only 0.3% achieved the fully-bound configuration. This effect appears to stem from a difference in the ensembles of extended and collapsed states which are controlled by the linker flexibility. Therefore, our simulations suggest that variations in the CaM linker’s propensity for alpha helical secondary structure can modulate the kinetics of target binding.

## 1. Introduction

**Figure 1:**
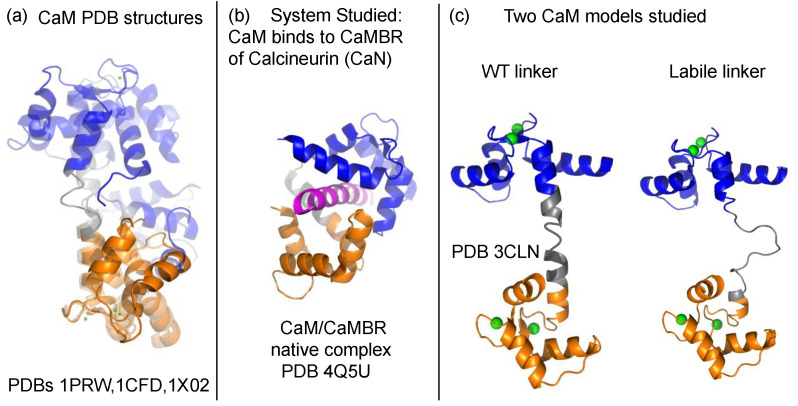
(**a**) Three CaM crystal structures aligned onto the C-domain to show the native flexibility of the linker [1]. The N- and C-domains are colored blue and orange, respectively. The central linker (residues 73 to 87) is colored gray. The PDBs of these structures are 1PRW, 1CFD, and 1X02. (**b**) The process of CaM binding to the CaM binding region (CaMBR) of calcineurin (CaN) was studied in this work. (**c**) Two CaM models, a WT linker with normal flexibility and a labile, highly-flexible linker were used.

Calmodulin (CaM) is a ubiquitously-expressed, 16.7 kDa globular protein that regulates hundreds of protein targets [1] in a Ca${}^{2+}$-dependent manner. Its primary sequence is identical across all vertebrates [2] and comprises two domains connected by a linker (Figure 1). How CaM maintains binding selectivity towards its targets with affinities ($K_{d}$) varying from nM [3] to μM [4] has been studied for decades [5,6,7,8,9,10,11,12,13,14,15,16]. These studies have generated valuable insights into factors contributing to its binding selectivity, which includes hydrophobic residues in targets that interact with CaM, CaM’s conformational heterogeneity at the binding surface, and its Ca${}^{2+}$-binding sensitivity. Of these, the flexibility of CaM’s linker is believed to play a prominent role in shaping the conformational ensemble it adopts [17,18] and thereby its regulation of target enzymes [19].

A key basis for CaM’s selectivity is attributed to the variety of binding modes it adopts when bound to its targets (reviewed in [20]). These include an extended conformation, a collapsed conformation in which its N- and C-domains wrap around the target, and intermediate configurations that permit CaM/target stoichiometries of 1:1, 2:2, or 2:1. Therefore, CaM’s ability to bind a variety of targets in part stems from the linker’s ability to assume different conformations [21,22]. Further, this flexible linker is believed to exert an entropic role in tuning target affinity. For instance, target-binding induced mobility changes in the linker residues correlated with the conformational entropy measured for the entire protein [22]. As another example, Katyal et al. [23] demonstrated that tethering the N- and C-CaM termini via a disulfide bond decreased the entropic penalty of binding by reducing the ensemble of thermodynamically-accessible states available to the linker.

While many thermodynamic details of CaM/target binding are increasingly understood, the protein’s binding kinetics remain enigmatic. It is believed that differences in the rates of CaM/target assembly provide a basis for CaM’s ability to selectively bind its targets [24]. One factor implicated in CaM’s target binding is the conformational flexibility endowed by the linker spanning the C- and N-terminal domains [17], but its effect on CaM/target binding kinetics has not been investigated. Understanding these molecular mechanisms could provide critical insights into the biological functions of this essential protein.

Molecular dynamics (MD) simulations have been used to probe the CaM/target binding processes [12,25]. One of the prominent challenges in these simulations is that most CaM targets are intrinsically disordered peptides (IDP) [26]. Such IDPs adopt stable secondary structures upon binding through ‘coupled binding and folding’ mechanisms that are case-dependent [27] and generally a combination of conformational-selection and induced-fit [28,29] binding. IDP binding mechanisms can unravel over microseconds and longer time scales that are generally inaccessible to conventional all-atom protein descriptions [30]. Efforts to bridge this limitation include adaptive sampling techniques such as goal-oriented sampling, weighted ensemble (WE), and coarse-grained techniques, which have been used to study the process of IDP/DNA binding [31], globular proteins binding [32] and protein/ligand binding [33]. Of these approaches, coarse-grained (CG) molecular dynamics simulations are attractive due to their sampling efficiency and ability to preserve important molecular details of IDP-binding [25,34,35,36,37]. Such simulations could provide important insights into the time-dependent nature of CaM/target assembly but have generally been limited to study mechanisms, but not kinetics [12,25].

In this study, we simulated the binding process of a model system, CaM, and the CaM binding region (CaMBR) of calcineurin (CaN), using a Martini CG model with explicit water molecules and ions. CaN is a ubiquitously expressed serine/threonine phosphatase in all human tissues [38] and regulates several biological processes [39] after being activated by CaM. Its binding to CaM is known to be rapid [40], which likely plays an important role in determining the kinetics of intracellular processes like gene regulation [41,42]. Further, experimental kinetic data are available for this system [40], which allows us to validate our computational results. The Martini CG simulations enabled us to sample native-like CaM/CaMBR complex structures from unbiased sampling over a range of ionic strengths to investigate electrostatic interactions and under different linker flexibilities. Based on these simulations, we propose a mechanistic basis for how CaM linker flexibility shapes the kinetics of target binding and its dependence on the solvent ionic strength.

## 2. Materials and Methods

### 2.1. Martini CG Simulations

We elected to use Martini CG given its advantages over ‘structure based’ models (SBM, also called G$\bar{o}$-like model [43]) and is well-suited for modeling the kinetics of target binding. The Martini CG mapping ratio is on average four atoms to one CG bead [44], while the commonly used G$\bar{o}$-based model is about ten nonhydrogen atoms to one bead [45]. This higher resolution permits more detailed descriptions of protein side-chain interactions with waters, which can impact protein mobility [46]. In addition, no reference-structure based potential is needed; therefore, the Martini CG potential is more easily extended to arbitrary proteins without refitting and the simulation time scale can be directly interpreted.

**Figure 2:**
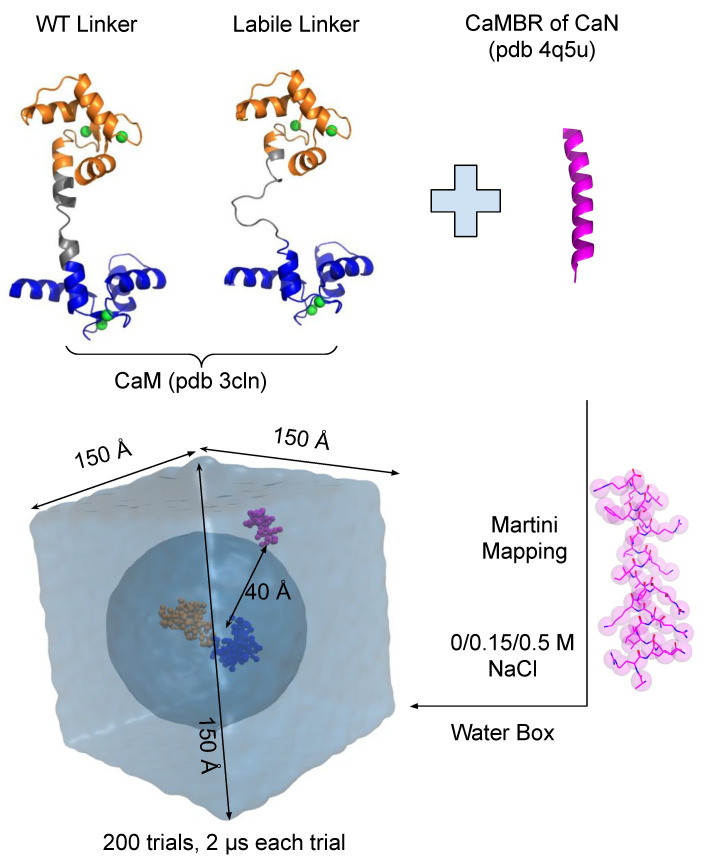
Martini CG setup. The all-atom CaM and CaMBR of CaN structures were from PDB 3CLN and PDB 4Q5U, respectively. Two CaM models with WT linker and labile linker were considered. After mapping the all-atom structures into Martini CG structures, the CaMBR was randomly placed around CaM with minimum distance >40 Å. The binding process was simulated by molecular dynamics without any further constraints imposed on the proteins.

We established a Martini CG model based on the extended CaM structure PDB 3CLN (Rattus rattus, CALM1_RAT, UniProtKB: P0DP29) [47]. The CaMBR of CaN was extracted from the CaM/CaMBR complex PDB 4Q5U (Homo sapiens, CALM1_HUMAN, UniProtKB: P0DP23) [48]. The two CaMs have identical amino acid sequences. Both CaM and CaMBR structures were used to construct the CG model with the Martini protein force field V2.2 [49,50]. Martini model’s time scale is four times faster than all-atom simulations [51]. Martini CG does not typically sample protein secondary structure thus it relies on user-provided secondary structure information to assign proper backbone parameters of bonds, angle, and dihedral terms [49]. Therefore, we calculated the secondary structure of the CaM and CaMBR via the DSSP program [52,53]. We constructed two CaM models, one with WT linker (residues 73–87) flexibility and one with a more labile and highly flexible linker (Figure 2). The WT linker was based on PDB 3CLN and had a 4-residue hinge (residues 78–81) that was predicted to assume turns (‘TTTT’) via DSSP. The labile linker was generated by short annealing MD simulations in vacuum using Amber [54] to remove the native secondary structure, and it was predicted to assume coils and bends (‘CCCSCCSSSCCCSSS’ where ‘C’ and ‘S’ refers to coil and bend, respectively) via DSSP. After defining the system, the CaMBR was randomly placed around CaM via the gmx insert-molecues command with a minimum distance between CaMBR and CaM of 40 Å to minimize bias. For each system, 200 trials were run with ionic strengths of 0, 0.15, and 0.5 M NaCl, respectively, in order to investigate how the electrostatic interactions between the CaMBR peptide and CaM affect the association process. The 0.15 M value corresponded to an ionic strength typical of the cell cytoplasm, while simulations at 0 M ionic strength nullified the screening of electrostatic interactions by solvent ions. Lastly, since experimental data were collected at 0.5 M [40], we simulated at 0.5 M as well. Elastic constraints within CaM’s N-/C-domains were introduced to maintain an open domain conformation [55], which was justified by our observations that the N-/C-domain conformations were unchanged after binding CaMBR (<2 Å RMSD). The Martini V2.0 ion force field was used to describe the NaCl ions added into the system and appropriate numbers were added to set the aforementioned ionic strengths. The system was solvated in a cubic water box with dimension of 150 Å per side using the gmx solvate command with pre-equilibrated waters from the Martini website. The standard Martini water model was used, in which four all-atom water molecules are combined into a single coarse-grained bead [56]. The system was first subject to 2000 steps of energy minimization using the steepest descent algorithm with constraints on the protein. The minimized system was equilibrated after being heated to 300 K over 6 ns. Constraints were imposed on the proteins during the heating stage. A 2 μs production run was initiated from the equilibrated system in the NPT ensemble using a 30 fs time step. The Berendsen temperature and pressure couplings were used to maintain 300 K and standard pressure. All simulations were performed using GROMACS version 2020.3 [57]. The back mapping from CG model to the all-atom model was done according to the procedure proposed in [58].

### 2.2. Analyses

The autoimage command from the CPPTRJ [59] program that centers and images the trajectory was used for periodic boundary condition treatment. The trajectory was then converted to PDB format with bonds added using the gmx trjconv command of Gromacs. The PDB format trajectory was used for all analyses and visualization. The contacts between CaMBR and CaM were calculated assuming that one contact represents any pair of beads that is within 5.5 Å. The CaMBR/CaM center of mass distance, RMSD to the native-like complex, and CaM’s radius of gyration ($R_{G}$) were calculated via the CPPTRAJ program. The CG structure of PDB 4Q5U served as a reference structure for the RMSD calculation. The trajectories were projected onto a plane according to the CaM/CaMBR center of mass distance and RMSD relative to the reference structure. The projection densities were estimated by a Gaussian kernel using the gaussian_kde from the scipy python library. The potential $E_{PMF}$ was estimated via Boltzmann inversion $E_{PMF}=-k_{b}T\ln\left(\rho /\rho_{min}\right)$ where $k_{b}$ is Boltzmann’s constant, *T* is temperature, ρ is the point density after projection and $\rho_{min}$ is the minimum density.

### 2.3. Association Rate Calculations Based on First Passage Time to Bound State

CaM and CaMBR were deemed bound when the structures assumed 50 or more contacts. We later justified that CaMBR/CaM complexes by this definition are thermodynamically favorable and located near the fully-bound state (see Section 3.3). The first passage time ($T_{fp}$) of reaching the bound state can then be used to estimate the association rate ($k_{a}$) [60,61]:

(1) $$\begin{array}{c} k_{a}=\frac{1}{T_{fp}\left[c\right]} \end{array}$$

where $\left[c\right]=1/\left(N_{A}V_{box}\right)$ is the equivalent concentration of one molecule in the simulation box with volume $V_{box}=3.375\times 10^{-21}L$ and $N_{A}$ is Avogadro’s constant. We used bootstrapping with replacement [62] to calculate the $T_{fp}$ from our simulations. For this, we randomly generated 1000 distributions of $T_{fp}$ values using the original 200 values generated by the simulations. The mean value of $T_{fp}$ was obtained from the bootstrapping results. Scripts for these analyses are available in https://github.com/huskeypm/pkh-lab-analyses (accessed on 8 May 2021) (2021-CaMBRmartini)

## 3. Results and Discussion

### 3.1. Linker Flexibility Impacts CaM/CaMBR Assembly

**Figure 3:**
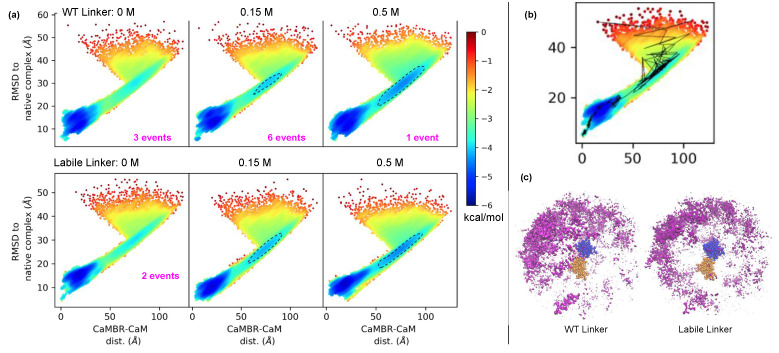
(**a**) Simulation trajectories projected onto a 2D plane: RMSD to native complex PDB 4Q5U and CaMBR/CaM center of mass distance. The dashed lines highlighted a redistribution of conformations into the bottle-neck region by increasing ionic strength. The numbers of binding events that led to native-like bound states are indicated in each panel. (**b**) An example trajectory of a binding event that led to a native-like bound state. (**c**) Distribution of the CaMBR about CaM calculated using structures at the bottle-neck region (RMSD ∼30 Å and CaMBR/CaM distance ∼75 Å.

To investigate how the CaM’s linker flexibility affects the binding process between CaM and the CaMBR of CaN, we performed extensive binding simulations using the Martini CG model with two CaM models that have WT and labile linker flexibility, respectively. The linker flexibility was controlled by setting its secondary structure property in the Martini model, hence a linker with a higher helical character was more rigid than a coil. For the target-free CaM crystal structure (PDB 3CLN), the linker (residues 73–87) was anticipated to have moderate flexibility. This was based on the DSSP result [52,53] suggesting that a four-residue ‘hinge’ spanning residues 78–81 assumed a ‘turn’-like (T) secondary structure, whereas the remainder was ascribed rigid alpha helical character. For the labile linker we assumed the entire linker consisted of coils and turns. To study the contributions of long-range electrostatic interactions in driving assembly [63,64], we performed 200 simulation trials at 0, 0.15, and 0.5 M ionic strength, respectively. In Figure 3 we show the projection of the trajectories onto two axes: (a) the CaMBR/CaM center of mass distance (b) the RMSD to native CaM/CaMBR complex crystal structure PDB 4Q5U to visualize the sampled conformational space. The population densities in the projected space were used to infer the potential of mean force ($E_{PMF}$) by inverting the Boltzmann equation.

Our simulations indicated that each system configuration exhibited significant sampling of a loosely-bound state, which was evidenced by the region of more negative $E_{PMF}$ values found where the RMSD was less than 20 Å and CaMBR/CaM distance was less than 35 Å. We noted that the loosely-bound states most prominently sampled a region modestly displaced from the reference crystal structure. We also observed a small number of binding events that yielded a native-like bound complex. The resulting structures had an average RMSD of 6.3 Å to the crystal structure of the CaMBR/CaM complex; although this value is perceived as relatively large for atomistic-resolution structures, by visual inspection (Figure S2) it was apparent that the binding poses closely resembled the native structure. Additionally, the ∼6 kcal/mol potential difference of this region relative to the unbound state indicated that the binding process was thermodynamically favorable. Interestingly, a bottle-neck was also apparent when the RMSD was within 25–40 Å and the CaMBR/CaM distance was between 60–100 Å for nonzero ionic strengths (see the dashed circles in Figure 3a). We attributed this bottle-neck region to two factors: (1) Electrostatic screening by ions and (2) constraints on CaM/CN alignment. We show in Figure S3 that the electrostatic potentials about CaM and CaMBR were prominent, complementary, and nonuniform. The largely negative electrostatic potential presented by CaM was expected to facilitate the binding of the positively-charged CaMBR peptide, as is well-established in other protein/protein complexes [63]. However, the negatively-charged potential can also stabilize off-target associations of the two proteins that could compete with binding to the native bound configuration. With increasing ionic strength, it appeared that the binding to the native bound configuration was disfavored, which manifested in a greater proportion of CaMBR/CaM binding poses that were off-target.

The nonuniform electrostatic potential also appeared to impose constraints on the CaMBR’s ‘angle of approach’ (Figure 3c). This was shown by the asymmetry in the distribution of CaMBR configurations about CaM. In other words, the nonuniform electrostatic potential of CaM and its highly labile conformational ensemble necessitated proper alignment of CaM with the CaMBR to facilitate productive binding. This mechanism of constraining the angle of approach has previously been observed in lysozyme/α-lactalbumin assembly [65]. In that study, it was shown that the binding process for two charged protein substrates had a strong preference for a narrow set of approach angles [65,66]. Further, the tendency for the two substrates to align decreased with increasing ionic strength. Therefore, we anticipated that the interplay of electrostatics and protein/protein alignment for CaM/CaMBR gave rise to a similar ‘funneled’ landscape that was consistent with the Lund et al. study [66].

CaM’s linker flexibility appeared to impact the degree to which the bottle-neck region was favored as the ionic strength was altered. In the WT linker CaM model, as the ionic strength was increased from 0.15 to 0.5 M, we observed a redistribution of states toward the bottle-neck region. For the labile linker, this change was much less pronounced. We believe this occurred because the labile linker allowed for a wider CaM/CaMBR angle of approach. This was evident based on the distribution of CaMBR configurations about CaM (Figure 3c) that fell within the bottleneck region. Notably, the CaMBR configurations were more diffusely and uniformly distributed about CaM, which suggested broader angles of approach were possible for the labile linker relative to the WT. We further discuss how this distribution shaped association rates between CaM and CaMBR in Section 3.3.

We also report for WT and labile linker CaM models the number of binding events that led to fully-bound configurations that were consistent with the reference crystal structure. We identified this bound state as the region near (0,0) of the native RMSD and CaMBR/CaM distance projection plane. For the WT linker CaM model, we observed ten events relative to only two for the labile linker model, out of ∼590 trials culminating in loosely-associated assemblies. This demonstrated that a highly labile linker significantly reduced the probability of achieving the native-like bound state. It is worth speculating that this reduced probability may have consequences in CaM’s ability to regulate its targets, namely by hindering target binding and subsequent activation. This interpretation is supported by observations that CaM can bind to its targets in different structural states, but only a subset of structural states can activate the target enzyme [67].

### 3.2. Linker Flexibility Determines the CaM Conformation Ensemble

To determine the basis for how the labile linker reduced the probability of achieving native-like bound states, we calculated the radius of gyration ($R_{G}$) of CaM just prior to forming the loosely-bound ensemble. In the WT linker CaM model, the $R_{G}$ distributions were fitted to a two-peak Gaussian distribution with maxima at 15.2 and 18.0 Å for all three ionic strengths. These two peaks corresponded to a highly compact structure and an extended structure, respectively. This distribution was consistent with the extended [47] and compact CaM [68] structures that have been experimentally-observed in the absence of a target. Transition path calculations have suggested that the extended CaM formation was slightly more favorable than the compact one with a ∼3–4 kcal/mol theromodynamic advantage and these two CaM states were separated by a ∼10 kcal/mol barrier [69]. Our $R_{G}$ data of the WT linker CaM model qualitatively agreed with these transition path results, as the integrated extended CaM probability density was greater than that of the compact states. Moreover, the probability densities did not significantly overlap.

**Figure 4:**
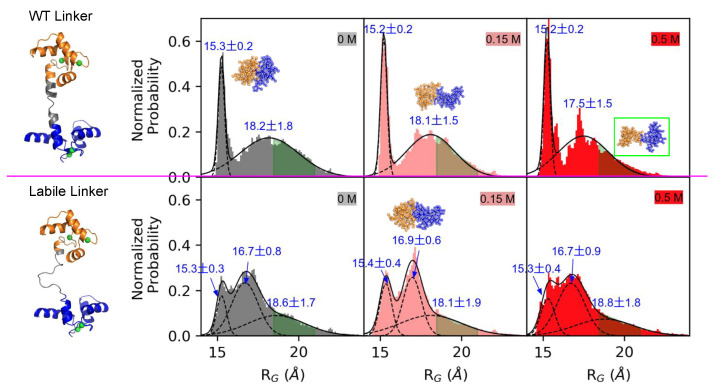
Radius of Gyration ($R_{G}$) of CaM at different ionic strengths before forming the loosely-bound state with CaMBR. The probability was normalized such that the area under the curve integrated to 1. The green shaded areas show the $R_{G}$s of all 12 binding events that led to native-like CaMBR/CaM complex with $R_{G}=19.7\pm 1.3$ Å. The three structures in the top row had $R_{G}$ values of 15.2, 18.0, and 19.7 Å, respectively. The structure in the bottom row was characterized by an $R_{G}$ value of 17.0 Å.

We contrast these data with those of the labile linker CaM model. For this configuration, a third peak emerged between the compact and extended distributions. The corresponding maxima were at 15.2, 17.0, and 18.0 Å, respectively. This additional probability density represented an intermediate state between the typical compact or extended conformations; the higher amplitude of which relative to the collapsed and extended states suggested that the intermediate state competed with the two extreme CaM configurations. In other words, the alpha helical linker of the WT model constrained the CaM conformational ensemble toward states that supported productive binding. We illustrated this by labeling in green the configurations amenable to CaMBR binding (green shaded areas in Figure 4). It was clear from this representation that the WT linker exhibited a higher percentage of states facilitating CaMBR binding relative to the labile linker.

### 3.3. Higher Linker Flexibility Attenuates the Sensitivity of the Association Rate to Ionic Strength

**Figure 5:**
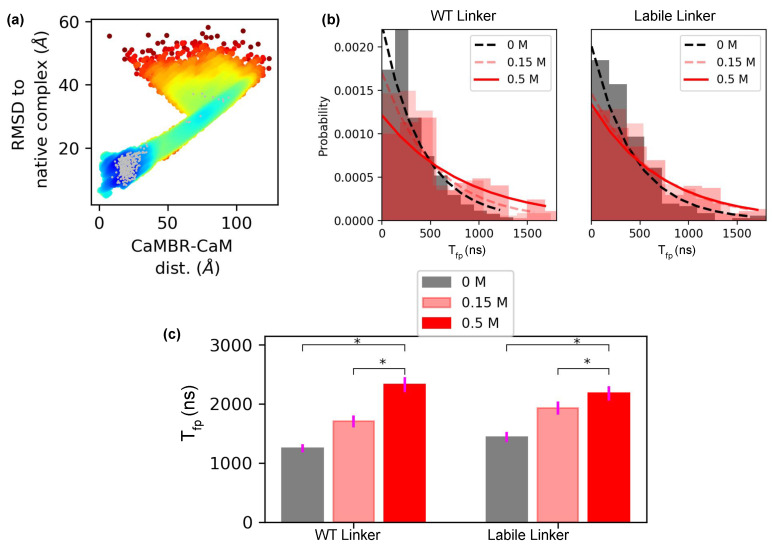
(**a**) Locations of loosely-bound state complexes, which were defined as those having at least 50 intercontacts between CaMBR and CaM. (**b**) Distribution of bootstrapped $T_{fp}$s from a sample consisting of 200 simulation trials (with replacement). The distribution was fitted to Aexp(−τx) to assess the convergence of the distribution of $T_{fp}$ values. (**c**) Average $T_{fp}$ after bootstrapping using 1000 samples. Because Martini CG’s time scale is four times faster than all-atom simulations [51], the $T_{fp}$ obtained from Martini CG has been multiplied by four to match all-atom time scale. The *p* values between the ionic strengths were calculated via Welch’s *t*-test, where * signified *p* < $1\times 10^{-3}$ and thus the difference of the means are significant.

We next related the markedly different conformation ensembles adopted by the WT and labile linkers to the kinetics of CaM/CaMBR assembly. For this, we computed a CaM/CaMBR association rate by assuming that binding was a one-step process from an unbound to a loosely-bound state. We based our assumption on observations that a fluorophore monitoring binding yielded a monophasic fluorescence signal versus time [40]. Specifically, Cook et al. used an acrylodan probe that reported changes in the proteins’ hydrophobicity and polarity as they assembled. Changes in the probes fluorescence signified CaM/CaMBR association, although they did not necessarily reflect formation of the fully-bound, native-like state. Hence, we defined the CaM and CaMBR loosely-bound state by the set of configurations that adopted 50 interprotein contacts. In Figure 5a we verified that the loosely-bound state complexes (in gray) were thermodynamically favorable ($E_{PMF}<0$) and located near the fully-bound state.

To estimate the association rate, $k_{a}$, we computed the first passage time $T_{fp}$ of reaching the loosely-bound state. In Figure 5b, we provide histograms denoting the distribution of $T_{fp}$ values following bootstrapping simulations. For both WT and labile linker cases, the $T_{fp}$s were exponentially distributed. Since the distributions rapidly converged to zero near the simulation limit of 2 μs, the simulations appeared to have sampled a sufficient number of association events for estimating $k_{a}$. The average $T_{fp}$ is plotted in Figure 5c. Both the WT and the labile cases exhibited similar dependence on ionic strength: increasing ionic strength increased $T_{fp}$, indicating that ions slowed down the association process by screening electrostatic interactions. However, when the ionic strength changed from 0.15 to 0.5 M, the WT case had a significantly larger $T_{fp}$ increase of ∼0.62 μs versus ∼0.25 μs for the labile case. This difference signified that WT linker CaM was more sensitive to electrostatic screening than the labile linker CaM.

Based on the estimated values of $T_{fp}$, we reported the calculated $k_{a}$ via Equation (1) in Table 1. The $k_{a}$ for the WT linker CaM model at 0.5 M ionic strength was estimated to be $8.7\times 10^{8}$ M^−1^ s^−1^ (Table 1), and within the diffusion-limited regime [63]. This compared favorably to the experimental value of $2.2\times 10^{8}$ M^−1^ s^−1^ [40]. Increasing concentrations of ions reduced the $k_{a}$ values for both CaM models. This dependence of $k_{a}$ on ionic strength was also observed in experimental measurements [40] and additionally confirmed by rigid-body Brownian dynamic simulations using isolated CaM domains and CaMBR (Figure S1) [64]. For the WT linker, increasing the ionic strength monotonically decreased the $k_{a}$, albeit more weakly for the labile linker case. We speculated that this weaker dependency was consistent with our observations that fewer labile linker configurations were confined to the bottle-neck region at high ionic strengths relative to the WT (Figure 3). This was expected as the thermodynamic stability of structures in the bottle-neck region likely increased the dwelling time of CaM/CaMBR structures in this state. In total, our simulations indicated that the rates of forming loosely assembled CaM/CaMBR configurations were comparable for the WT and labile configurations and were strongly driven by electrostatic interactions. Importantly, the WT configuration strongly favored configurations that led to fully-bound assemblies relative to the labile CaM.

Although association events were frequent, we did not observe any dissociation events upon assembly of the CaMBR and CaM complex. This was not surprising, given that $k_{off}$ values can be considerably slower than the corresponding association rates [70]. For our system, the $\Delta G_{bind}$ was assumed to be 11 kcal/mol [26]. Based on our computed value of $k_{a}$ = $8.7\times 10^{8}$ M^−1^ s^−1^ at 0.5 M ionic strength, this implied a $k_{off}$ value of 7.6 s^−1^. Conventional MD simulations have been used to model complexes with fast dissociation rates [61,71], but generally biased sampling simulations are often necessary to simulate dissociation processes occurring at timescales in the seconds or longer [72]. Such simulations were beyond the scope of this study.

## 4. Limitations

We note a few limitations of our study that could be refined in subsequent investigations. In our Martini CG model setup, two assumptions were made: (1) the N and C domains of CaM were assumed to be rigid and (2) the secondary structure of CaMBR of CaN was fixed as an alpha helix. The first assumption was reasonable as our comparisons of the crystal structures for CaM and CaMBR/CaM complex indicated that the structures did not significantly differ (<2 Å RMSD). The latter assumption could mask any conformational rearrangement that the CaMBR may contribute to an “induced fit” binding mechanism. However, we anticipated that this limitation should only have a marginal impact on our results reported here. This was motivated by the notion that IDP binding mechanisms are generally characterized by a “couple binding and folding” paradigm comprising “conformational selection” and “induced fit” [29,74]. Our previous studies [64] demonstrated that when the CaMBR was simulated in the absence of CaM, it adopted partially-folded alpha helix structures (68.7% of the ensemble fell within 7–9 Å RMSD of a perfect alpha helix) in a manner consistent with “conformational selection”, as opposed to “induced fit”.

In our association rate calculations, we assumed a one-step binding process based on experimental measurements. However, a two-step binding process consisting of (1) an unbound to loosely-bound (encounter complex) assembly and (2) a loosely-bound to native bound assembly through structural reorganization, was also feasible. This mechanism has been observed in other IDP binding processes [75,76,77]. To investigate this potential mechanism, experimental strategies that can monitor both the encounter complex and bound states are necessary. Such a strategy has been applied to melittin binding to CaM [78], for which a dansyl probe was used as a fluorescence resonance energy transfer (FRET) acceptor from a melittin tryptophan residue. The approach indicated that the fluorescence signal was biphasic in time, therefore suggesting that there was a fast rate of encounter-complex assembly, followed by a slower structural reorganization into the native complex. Analogous experimental studies for CaN binding would be helpful in elucidating this potential mechanism.

Lastly, although the CaM sequence is identical across all vertebrates [2], its conservation can be considerably lower in invertebrates and plants, which would likely impact its binding kinetics to CaN and other targets [79].

## 5. Conclusions

CaM’s flexible linker plays an important role in target binding. In this study, we utilized the Martini coarse grained (CG) model to simulate the binding process between CaM and a representative protein target, the CaMBR of CaN. Our simulations examined the fully-bound native-like CaMBR/CaM complexes under normal and highly-flexible linker conditions. The latter exhibited few events that led to fully-bound configurations, suggesting that a higher linker flexibility reduced the probability of achieving productive binding. Our data indicated that the enhanced linker flexibility disturbed CaM’s conformational ensemble in a manner that limited the sampling of states compatible with fully-bound assemblies. This limited sampling could likely slow the rate and efficiency of CaM binding to regulate targets [67], such as the CaN phosphatase considered in our study.

Our results further highlight the importance of electrostatic interactions in driving CaMBR and CaM assembly. Notably, the nonuniform electrostatic potential along the CaM solvent exposed surface imposed constraints on angles of approach leading to target binding. We demonstrated that this stereospecific constraint was more significant for the WT linker relative to the highly labile configuration, suggesting that linker flexibility shapes the assembly mechanism. Further, these properties influenced the kinetics of CaM/CaMBR assembly and their sensitivity to changes in ionic strength. In summary, our study emphasized the important role of CaM’s linker properties in shaping the thermodynamics and kinetics of CaM/target assembly. Our findings could therefore shed light into how CaM target regulation could be impacted by modulation of CaM’s linker properties, as might be expected for the linker-localized CaM missense mutations (M77I and S82R) [80] and post-translational modifications, for instances, phosphorylation at sites T80 and S82 [81].

## Figures and Tables

**Table 1:** The mean first passage time ($T_{fp}$) to form the loosely-bound state (Figure 5) and the corresponding $k_{a}$ calculated via Equation (1).

	WT Linker			Labile Linker		
	0 M	0.15 M	0.5 M	0 M	0.15 M	0.5 M
*T*${}_{fp}$ (ns)	1247.78 ± 0.05	1708.28 ± 0.06	2326.98 ± 0.05	1441.32 ± 0.06	1930.28 ± 0.06	2178.93 ± 0.06
$k_{a}$ (10${}^{8}$ M${}^{-1}$ s${}^{-1}$, 300 K) ^*a*^	16.29 ± 0.92	11.90 ± 0.70	8.73 ± 0.46	14.10 ± 0.85	10.53 ± 0.60	9.33 ± 0.53
Expt. $k_{a}$ (10${}^{8}$ M${}^{-1}$ s${}^{-1}$, 310 K) ^*b*^			2.2 ± 0.44			

^*a*^ The calculated $k_{a}$s are within the diffusion-limited regime as they are >$1\times 10^{6}$ M^−1^ s^−1^ [63]. ^*b*^ The 10 K temperature difference between experiments and simulations does not affect the comparison because this small temperature has negligible impact on measured kinetics [73].

## Data Availability

The simulation trajectories are available upon reasonable request.

## Supplementary Materials

The following are available online at https://www.mdpi.com/article/10.3390/ijms22094990/s1, Figure S1: Association rates between CaM and the calcineurin CaMBR from Brownian dynamics (BD) simulations, Figure S2: Superimposition of simulated fully-bound native-like CaM/CaMBR complex structure with the crystal complex structure, Figure S3: Electrostatic potentials of collapsed and extend CaM structures and the CaMBR helix.

## Abbreviations

The following abbreviations are used in this manuscript:

CaM	Calmodulin
CaN	Calcineurin
CaMBR	CaM Binding Region
CG	Coarse-Grained
MD	Molecular Dynamics
COM	Center of Mass
RMSD	Root Mean Square Deviation
