# Supplementary material for: Assessing the Role of Calmodulin’s Linker Flexibility in Target Binding
Source: Int J Mol Sci. 2021 May 8;22(9):4990. doi: 10.3390/ijms22094990 (PMC8125811; doi:10.3390/ijms22094990)
Supplement: Supplementary file 1 [file ijms-22-04990-s001.zip › ijms-1192213-supplementary.pdf]

Supplementary Information (SI): Assessing the Role of Calmodulin's Linker Flexibility in Target Binding

Bin Sun[1] and Peter M. Kekenes-Huskey[*,1]

[1] Department of Cell and Molecular Physiology, Loyola University Chicago, Maywood, IL, USA 60153
* Corresponding author. E-mail address: pkekeneshuskey@luc.edu (P. Kekenes-Huskey)

May 4, 2021

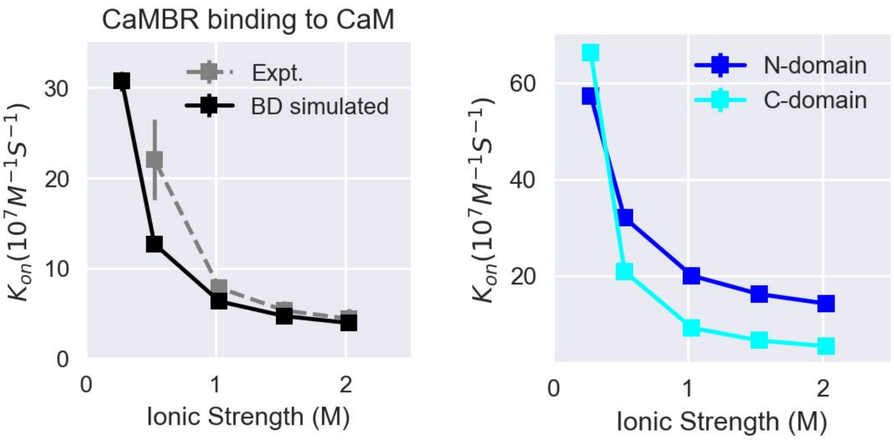

Figure S1: Association rates between CaM and the calcineurin CaMBR from Brownian dynamics (BD) simulations using the BrownDye package [1] (left panel). The binding of peptide to CaM's N- and C- domain are treated as independent events [2] (right panel). The overall association rate between CaM and CaMBR is then approximated as $1/k_{overall} = 1/k_n + 1/k_c$ [2], where k_n and k_c correspond to CaMBR association with the N and C domains, respectively. The BD simulation parameters were fitted to reproduce the experimentally-determined association rate at 1 M ionic strength .

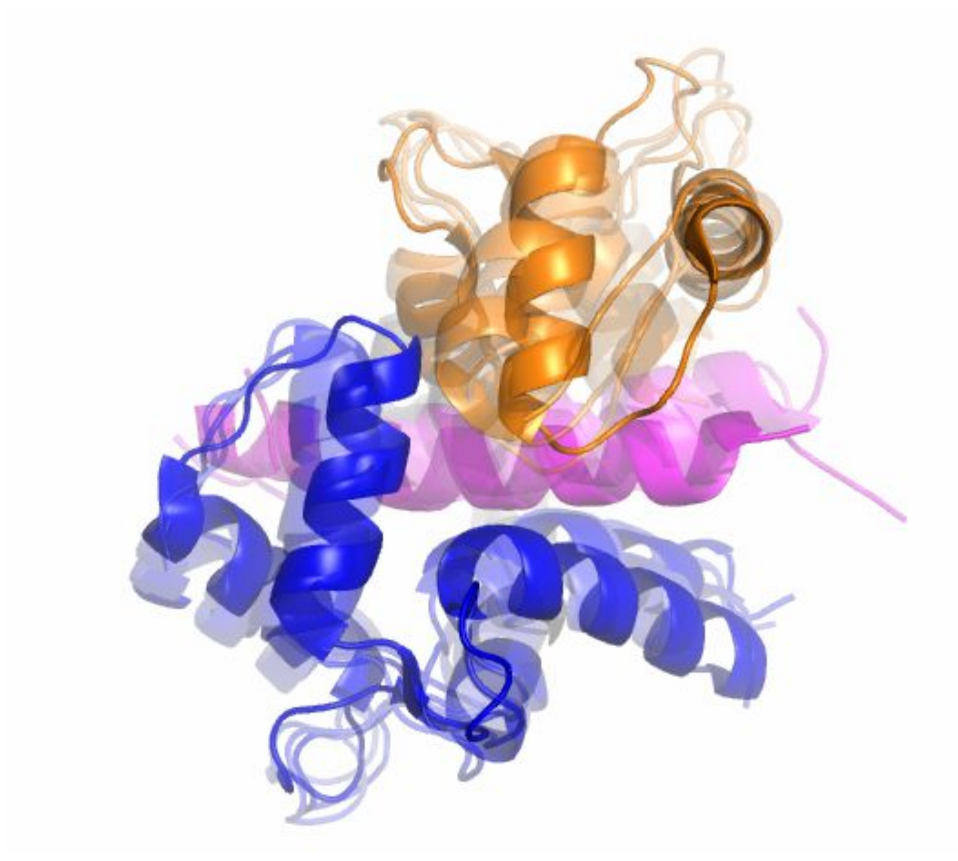

Figure S2: Superimposition of simulated fully-bound native-like CaM/-CaMBR complex structure with the crystal complex structure PDB 4Q5U. The simulated structures are shown as transparent cartoons. .

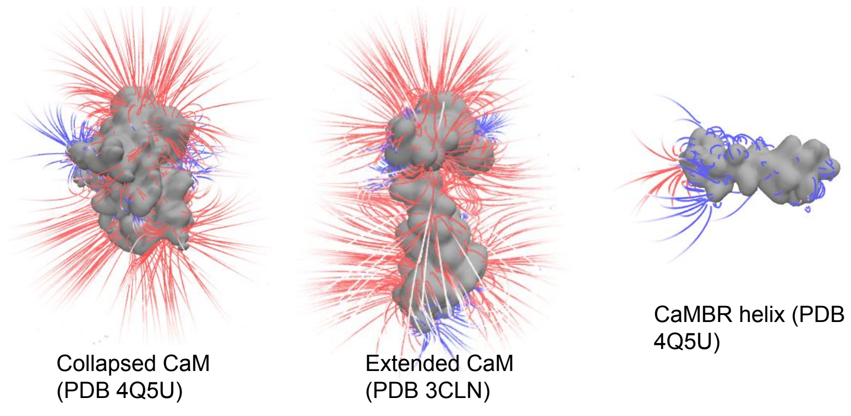

Figure S3: Electrostatic potentials (-0.2 to 0.2) of collapsed and extend CaM structures and the CaMBR helix. The electrostatic potentials were calculated via the APBS program at 0.15 monovalent ionic strength. The pqr files of each structure were generated using the pdb2pqr program with amber force field used.

References

[1] G. A. Huber and J. A. McCammon. "Browndye: A software package for Brownian dynamics". In: *Computer Physics Communications* 181.11 (2010), pp. 1896–1905.

[2] B. Sun et al. "Electrostatic control of calcineurin's intrinsically-disordered regulatory domain binding to calmodulin". In: *Biochimica et Biophysica Acta - General Subjects* 1862.12 (2018), pp. 2651–2659. ISSN: 18728006.
